# Development of genic SSR marker resources from RNA-seq data in *Camellia japonica* and their application in the genus *Camellia*

**DOI:** 10.1038/s41598-021-89350-w

**Published:** 2021-05-10

**Authors:** Qingyuan Li, Xiaojun Su, Huanhuan Ma, Kebing Du, Min Yang, Baolin Chen, Shao Fu, Tianjing Fu, Conglin Xiang, Qing Zhao, Lin Xu

**Affiliations:** 1 Forestry and Fruit Tree Research Institute, Wuhan Academy of Agricultural Sciences, Wuhan, 430075 China; 2 College of Horticulture and Forestry Sciences, Huazhong Agricultural University, Wuhan, 430070 China; 3 College of Plant Science and Technology, Huazhong Agricultural University, Wuhan, 430070 China

**Keywords:** Genetics, Plant sciences

## Abstract

*Camellia* is a genus of flowering plants in the family Theaceae, and several species in this genus have economic importance. Although a great deal of molecular makers has been developed for molecular assisted breeding in genus *Camellia* in the past decade, the number of simple sequence repeats (SSRs) publicly available for plants in this genus is insufficient. In this study, a total of 28,854 potential SSRs were identified with a frequency of 4.63 kb. A total of 172 primer pairs were synthesized and preliminarily screened in 10 *C. japonica* accessions, and of these primer pairs, 111 were found to be polymorphic. Fifty-one polymorphic SSR markers were randomly selected to perform further analysis of the genetic relationships of 89 accessions across the genus *Camellia*. Cluster analysis revealed major clusters corresponding to those based on taxonomic classification and geographic origin. Furthermore, all the genotypes of *C. japonica* separated and consistently grouped well in the genetic structure analysis. The results of the present study provide high-quality SSR resources for molecular genetic breeding studies in camellia plants.

## Introduction

*Camellia* is a genus of flowering plants in the family Theaceae, which is widely distributed in Southeast Asia, from the Himalayas to Japan and from southern China (Guangxi and Yunnan) to Java and Sumatra^1^. Several species in the genus *Camellia* have economic importance. Species such as *C. japonica*, *C. reticulata*, *C. saluenensis* and *C. sasanqua*, are well-known as camellias with attractive flowers. The young leaves of the important economic species *C. sinensis* are used to produce tea. A few species such as *C. oleifera* and *C. semiserrata* are used to produce high-quality edible and pharmaceutical seed oil^1,2^.

*Camellia* is the largest genus in the family Theaceae. This genus is believed to comprise more than 300 species^3^, indicating genetic instability and the high outbreeding nature of the genus. Ornamental camellias (chahua in Chinese) have been grown in China for 2,000 years^4^. The common camellia was introduced to Japan over 1,000 years ago^4^. Ornamental camellias were brought to Europe and the Americas in the late 1870s^5^ and are now popular flowering and landscaping shrubs in many regions worldwide^1^. Currently, there are more than 3,000 cultivated varieties of ornamental camellia worldwide^1^. Due to natural and artificial interspecific hybridization *Camellia*, this genus exhibits important taxonomic and systematic conflicts^6^.

Traditionally, the breeding of camellia plants was based on hybridization among species and cultivars and the phenotypic selection of novel or improved offspring^7,8^. Although traditional breeding still plays a crucial role in the quality improvement of *Camellia* plants, it is limited by the long selection term and tremendous resources required in the breeding of new varieties. Marker assisted selection (MAS) is potential to accelerate plant breeding and avoids the problems associated with traditional plant breeding by improving the selection criteria from phenotypes to genes, thus saving time and resources^9,10^. Moreover, molecular markers are not restrained to environmental regulations, and are not affected by the conditions that the plants are grown and detectable at all stages of plant growth^11^.

Simple sequence repeats (SSRs) are genomic fragments that composed of tandemly repeated units of 1–6 nucleotide sequence motifs flanked by unique sequences^12^. SSR markers are widely used in plant genetic improvement and MAS breeding. Due to their high polymorphism, codominant inheritance, and wide distribution traits throughout the genome^12,13^, SSRs have been extensively used in genetic linkage map construction, genetic identification, genetic diversity analyses and fingerprinting construction^14–17^.

A great deal of effort has been made to develop SSR markers in the genus *Camellia* in the past decade. Based on 454-sequencing data, 36 polymorphic expressed sequence tag (EST)-SSR markers have been developed in tea plant (*C*. *sinensis*)^18^. By using high-throughput Illumina RNA sequencing data, 431 polymorphic SSR markers were developed, and a consensus SSR-based linkage map was constructed that covered 1,156.9 cM with 237 SSR markers distributed in 15 linkage groups in tea plant^19^. Based on transcriptome data, 450 polymorphic SSR markers were developed, and 406 were successfully added to genetic linkage maps of tea plant^20^. Polymorphic SSR markers were developed via genome sequence analysis of tea plant and then used for genetic studies and fingerprinting construction^13,21^. Several efforts have also been made to develop SSR markers in ornamental camellias and oil camellias^22–27^.

However, the number of SSRs publicly available for plants in the genus *Camellia* is insufficient for some applications, such as the construction of high-resolution linkage maps, genome comparative mapping, genetic studies, and increasing marker density in specific map regions. Therefore, more efforts are still needed to develop SSR markers for further progress in camellia genetic and genomic studies.

In our previous study, we obtained approximately 1,006 million RNA-Seq reads by deep sequencing of the ornamental camellia *C. japonica* leaf transcriptome, using the Illumina sequencing platform^28^. These sequence data provide a good resource for the development of genic SSR markers. In the present study, we developed a set of novel genic SSR markers based on de novo transcriptome sequencing of *C. japonica*, determined transferable and polymorphic SSR markers for other *Camellia* species, and revealed the genetic relationships and genetic structure of *Camellia* germplasms. The results of this study will provide essential information for further studies in camellia plants such as taxonomic studies, diversity analysis, MAS and SSR-based genetic linkage mapping.

## Results

### Characteristics of genic SSRs in the *C. japonica* leaf transcriptome

SSRs were highly abundant in the assembled *C. japonica* leaf transcriptome. In total, 28,854 potential SSRs with a minimum of five repetitions for all motifs were identified from 24,368 contigs, representing 11.74% of the total 207,592 unigenes^28^ generated by Illumina sequencing. The frequency of occurrence of SSR loci was one in every 4.63 kb (221.17 SSRs/Mb) of the unigene sequence. The length of SSRs ranged from 12 to 279 bp, with an average of 20.54 bp.

Incidences of different repeat types and frequencies for each motif were evaluated based on the repeat unit number (Table 1). SSRs existed primarily as dinucleotide repeats and trinucleotide repeats, accounting for 97.59% of all SSRs. Dinucleotide repeats (74.56%) were the most abundant repeat unit, followed by tr- (23.08%), tetra-(2.04%), hexa-(0.19%) and pentanucleotides (0.11%), with the repeat unit number from 5 to 18. Most (99.4%) of the motifs had 5–10 repeats, while motifs with more than 10 repeat were rare (0.59%). Among the identified SSRs, AG/CT was the most common type of all dinucleotide repeat motifs (62.19%) (Supplementary Fig. 1a). The predominant trinucleotide repeat motifs were AAG/CTT and AAT/ATT, which accounted for 24.94% and 19.56% of the SSRs, respectively (Supplementary Fig. 1b). For tetranucleotide repeats, the most frequent motif was AAAT/ATTT (31.30%), followed by AAAC/GTTT (13.37%) and AAAG/CTTT (11.68%) (Supplementary Fig. 1c). These results may reflect the AG/CT-rich nature of the *C. japonica* transcriptome.

**Table 1:** Frequencies of different SSR repeat motif types observed in the *C. japonica* leaf transcriptome.

SSR motif	Repeat number								Percentage
	5	6	7	8	9	10	11–18	Tatal	(%)
Dinucleotide	0	6682	5187	4760	3599	1121	167	21,516	74.51
Trinucleotide	4022	1901	715	20	0	0	2	6660	23.08
Tetranucleotide	532	52	4	0	0	2	1	591	2.05
Pentanucleotide	22	5	2	0	1	0	1	31	0.18
Hexanucleotide	27	20	5	2	1	0	1	56	0.19
Total	4603	8660	5913	4782	3601	1123	172	28,854	100.00
Percentage (%)	15.95	30.01	20.49	16.57	12.48	3.89	0.60	100.00	

### Functional annotation of SSR-containing unigenes

**Figure 1:**
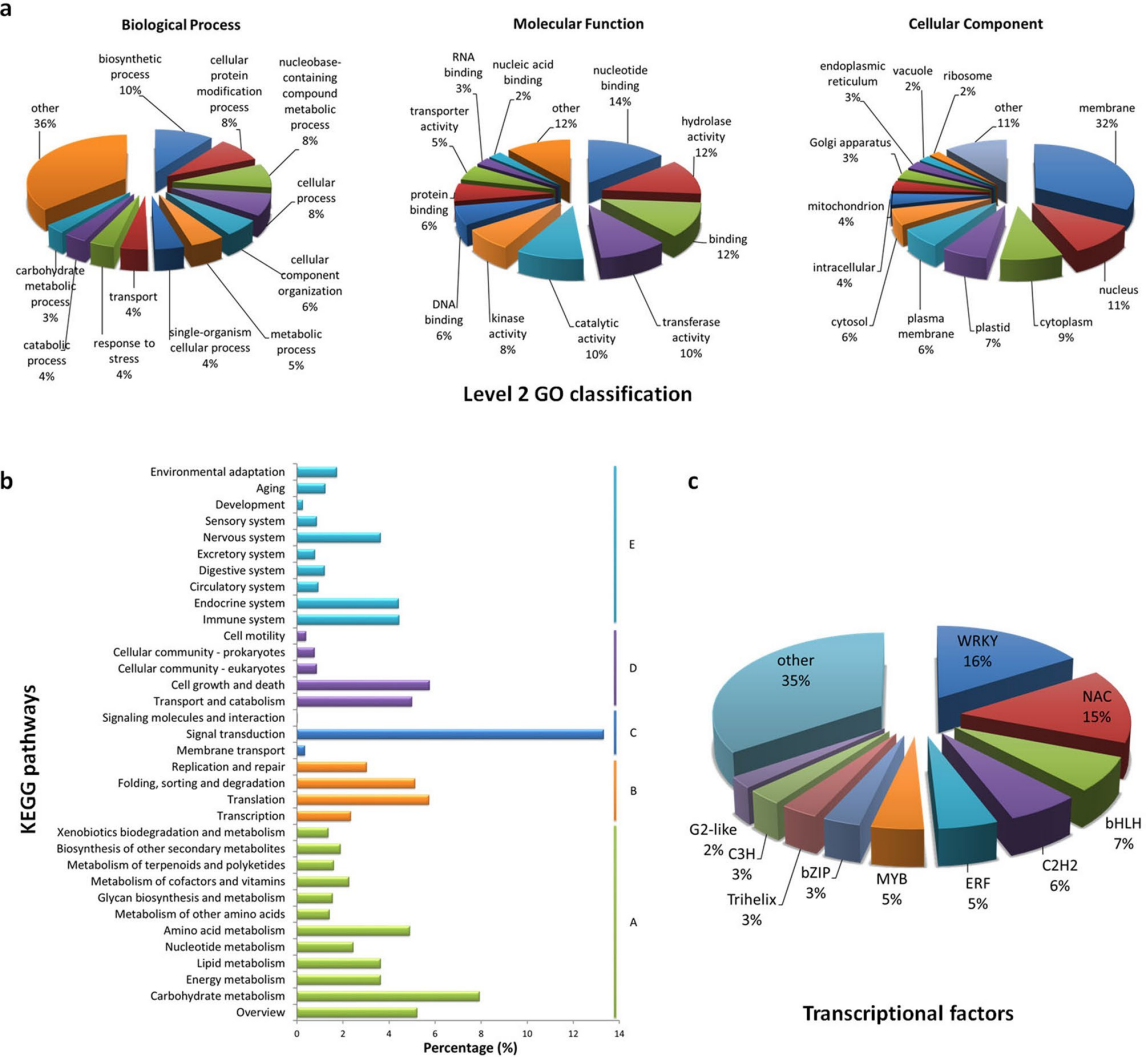
Functional annotation of SSR-containing unigenes in *C. japonica*. (**a**) Level 2 GO classification of the annotated SSR-containing unigenes. (**b**)KEGG classification of the annotated unigenes. (**c**) Putative TF gene families among the annotated SSR-containing unigenes.

To explore the potential function of these SSR-containing unigenes we used Gene Ontology (GO) assignments to classify the predicted *C. japonica* genes. A total of 24,214 SSR-containing unigenes were assigned to three major functional categories (Biological Process, Molecular Function and Cellular Component) (Fig. 1a). In the Biological Processes category, ‘biosynthetic process’, ‘cellular protein modification process’, ‘nucleobase-containing compound metabolic process’, and ‘cellular process’ were the top four GO terms. In the Molecular Function category, the unigenes were predominantly assigned to the ‘nucleotide binding’, ‘hydrolase activity’ and ‘binding’ groups. In the Cellular Component category, the unigenes were frequently assigned to ‘membrane’, ‘nucleus’, ‘cytoplasm’ and ‘plastid’.

To identify the biological pathways associated with the annotated SSR-containing unigenes, we annotated the unigenes to the reference pathways in the Kyoto Encyclopedia of Genes and Genomes (KEGG) using KeggArray software, and 4,325 SSR-containing unigenes were assigned to five specific pathways, including ‘Metabolism’, ‘Genetic Information Processing’, ‘Environmental Information Processing’, ‘Cellular Processes’, and ‘Organism Systems’ (Fig. 1b). Among these pathways, the ‘signal transduction’ cluster represented the largest group, followed by ‘carbohydrate metabolism’ and ‘cell growth and death’.

A total of 1,015 putative transcription factors (TFs) were identified in the annotated SSR-containing unigenes. These TFs were classified into 51 common families based on the classification of their Arabidopsis homologues (Supplementary Table 1). Among these families, the WRKY family was the largest group (161, 16%), followed by the NAC family (149, 15%), the bHLH family (71, 7%), the C2H2 family (64, 6%) and ethylene-responsive TF (ERF) (52, 5%) (Fig. 1c).

### Development and validation of genic SSR markers

**Figure 2:**
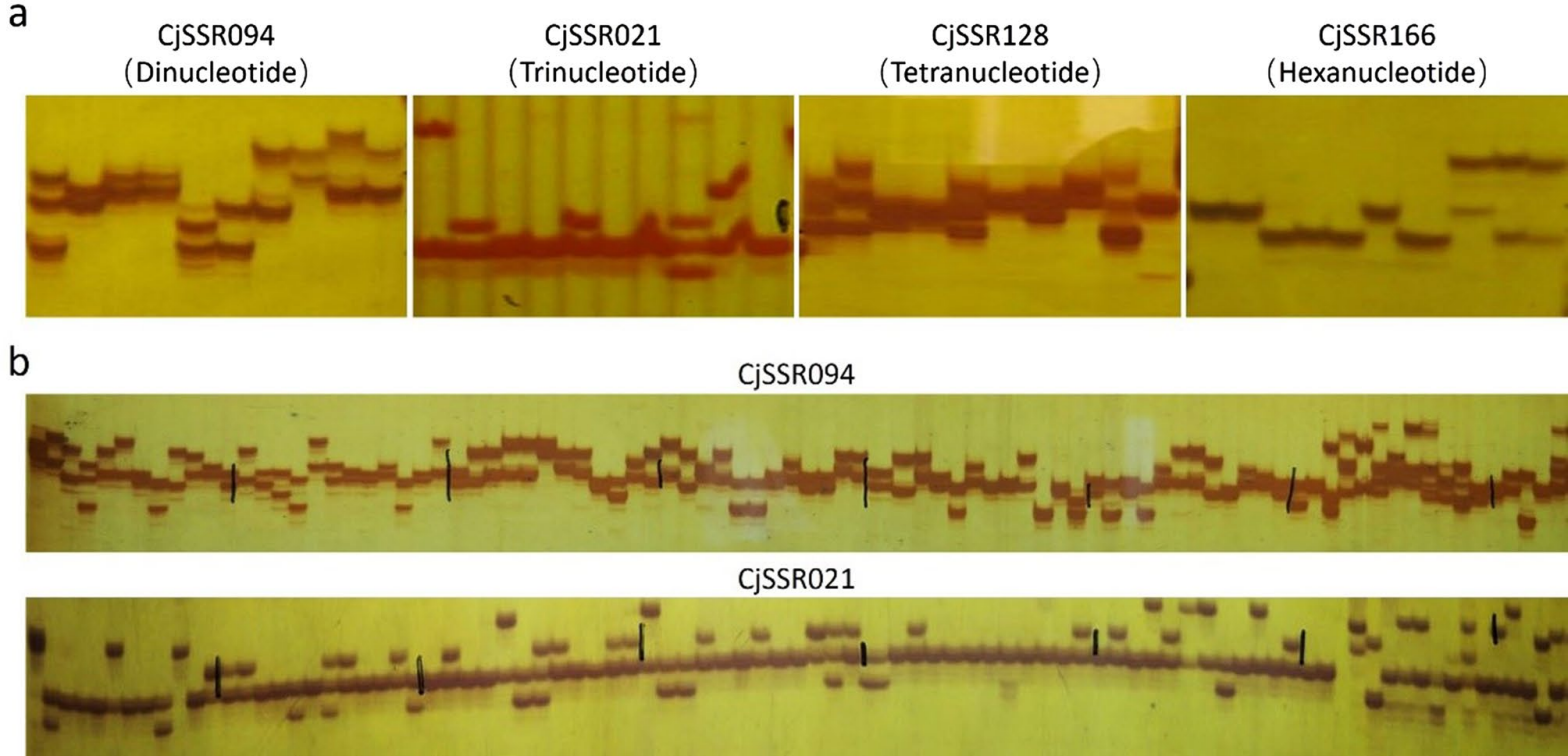
PAGE results of marker detection and population analysis. (**a**) Marker detection in 10 *C. japonica* accessions. (**b**) Population analysis in 89 accessions across genus *Camellia*. CjSSR094, CjSSR021, CjSSR128 and CjSSR166 were chosen from SSR markers with di-, tri-, tetra-, and hexanucleotide repeat, respectively.

A total of 24,368 SSR-containing unigenes were employed for primer design, from which 12,194 (50.04%) unigenes could be successfully used for SSR primer development. A total of 13,323 SSR primers were developed. After screening by e-PCR, 8,442 SSR primers were inferred to have unique amplification sites in the *C. japonica* leaf transcriptome. Of the 8,442 primer pairs, 172 were selected for primer synthesis. The 172 markers were tested for amplification using a panel of 10 *C. japonica* accessions (Fig. 2a), the details which are available in Supplementary Table 2. Of these SSR markers, 30 (17.44%) amplified smear or nonspecific products, and 3 (1.74%) generated no products in any of the *C. japonica* accessions. A total of 139 (80.81%) SSR markers yielded amplification products in the 10 accessions, of which 111 (64.53%) exhibited polymorphism. Among these polymorphic markers, trinucleotide repeats were the most abundant (66, 59.46%), followed by di- (30, 27.03%), tetra- (14, 12.61%) and hexanucleotide (1, 0.9%), whereas no polymorphic markers were identified in pentanucleotides (Table 2).

**Table 2:** Frequencies of different SSR motif types and the PIC values in the polymorphic genic SSR markers.

SSR motif	Number	Percentage	PIC value
		Among polymorphic markers (%)	
Dinucleotide	30	27.03	0.63
Trinucleotide	66	59.46	0.57
Tetranucleotide	14	12.61	0.58
Hexanucleotide	1	0.90	0.37
Total	111	100.00	0.59

A total of 495 alleles were identified across these 111 polymorphic genic SSR loci, and the number of alleles ranged from 1 to 12 with an average of 4.46 alleles per locus. To evaluate and characterize these polymorphisms so that they can potentially be used for assessing molecular diversity or genetic structure analysis, the polymorphism information content (PIC) values of these polymorphic primers were calculated. The PIC values of the polymorphic markers ranged from 0.15 to 0.86, with a mean value of 0.59. The PIC values of the polymorphic markers with di-, tri-, tetra-, and hexanucleotide repeat were 0.63, 0.57, 0.58 and 0.37, respectively (Table 2). The sequences of these informative newly developed genic SSR primers and other major information are shown in Supplementary Table 3.

In total, 51 genic SSR markers, which demonstrated polymorphic in these 10 *C. japonica* accessions, were randomly selected and further used to assess their transferability in 12 other accessions across 8 species in the genus *Camellia*, including *C. sasanqua*, *C. chuongtsoensis*, *C. rosthorniana*, *C. nitidissima*, *C. oleifera*, *C. reticulata*, *C. sasanqua* and *C. sinensis*. In contrast to five primer pairs, CjSSR014 and CjSSR021, that could not amplify any fragments in *C. sinensis*, CjSSR048 and CjSSR101, that could not amplify fragments in two *C. sasanqua* accessions, CjSSR098, that could not amplify fragments in *C. azalea* cultivar 'Nanyue Hongxia', the other forty-six primer pairs successfully amplified PCR products in all the tested accessions. Thus, the transferability rate across 8 species ranged from 94.12% to 100%, with an average transferability rate of 98.69% (Supplementary Table 4).

### Cluster analyses of natural accessions in the genus *Camellia*

**Figure 3:**
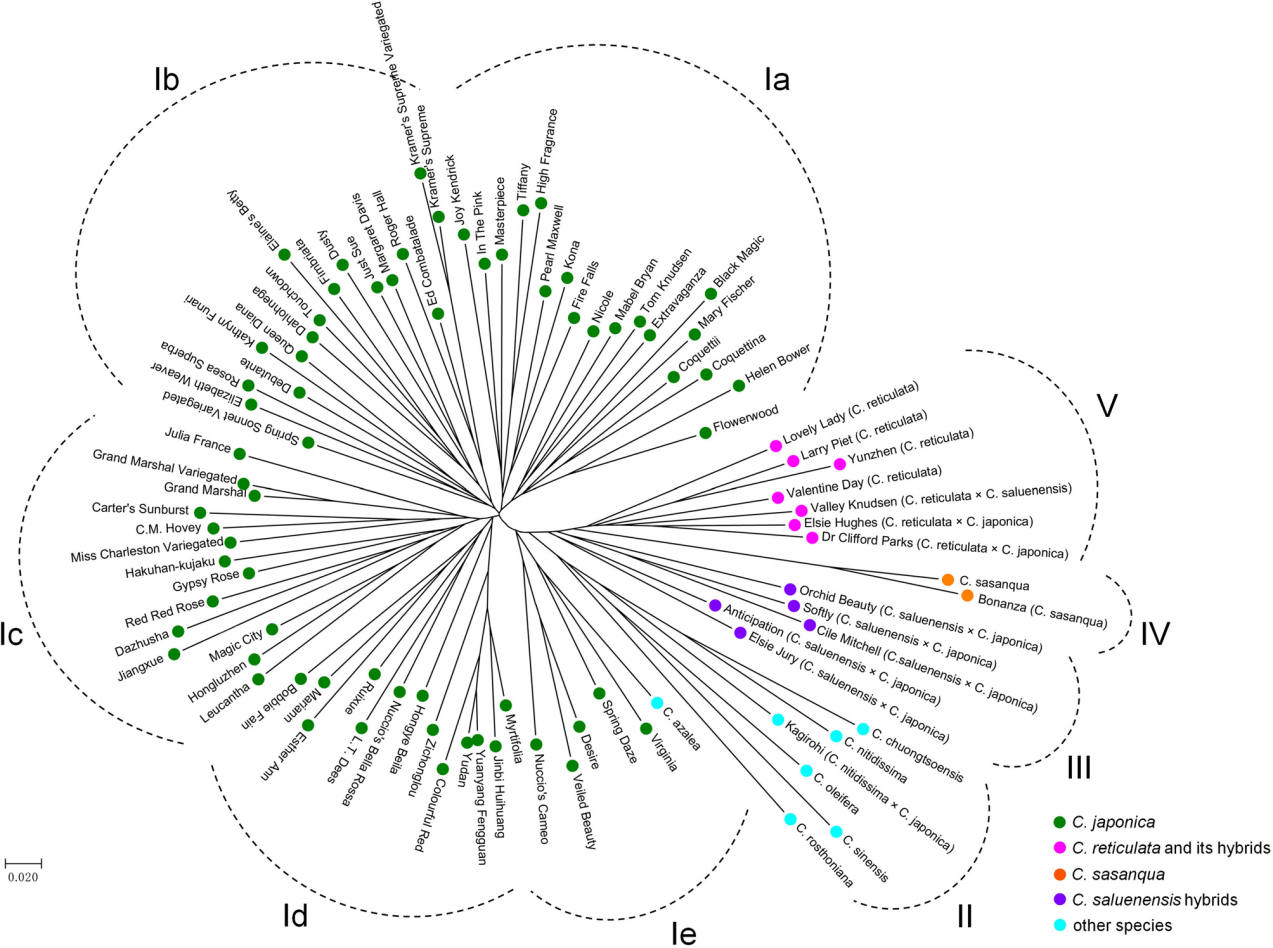
NJ dendrogram showing genetic relationships among the 89 camellia accessions. Accessions labeled according to the genetic background. Groupings of genotypes that agreed with groupings based on taxonomic classification.

To verify the applicability of the newly developed genic SSR markers, a total of 89 accessions across the genus *Camellia* (Supplementary Table 5) were genotyped by the above 51 genic SSR primers (Fig. 2b). In total, 503 alleles were obtained among these natural population, with 9.9 alleles per SSR on average, which was much higher than the previous studies^13,15,23,27^, implying that these 51 SSRs harbored abundant variation loci of Camellia. Therefore, these 51 SSRs as core molecular markers had typical and highly representative alleles. Based on the genetic distance results, several clusters were visible in the neighbor-joining (NJ) dendrogram. To simplify the description of the results, we distinguished five major clusters, I to V, by separating the longest branch into five branches. Cluster I comprised the accessions from *C. japonica* and was further divided into five subclusters: Ia to Ie. The accessions from *C. saluenensis* hybrids were grouped in cluster III. Cluster IV contained two accessions from *C. sasanqua*. The accessions from *C. reticulata* and its hybrids were closely clustered in cluster V. Accessions collected from other species in the genus *Camellia* were located in cluster II (Fig. 3). This result showed groupings of genotypes that agreed with groupings based on taxonomic classification and geographic origin. Notably, several accessions known to have similar genetic backgrounds were closely clustered and clearly distinguished with each other in the dendrogram, such as ‘Coquettii’ and ‘Coquettina’, ‘Yudan’ and ‘Yuanyang Fengguan’, and ‘Grand Marshal’ and ‘Grand Marshal Variegated’ (Fig. 3). The above results implied that the newly developed genic SSR markers are powerful for determination relationship of genotypes.

**Figure 4:**
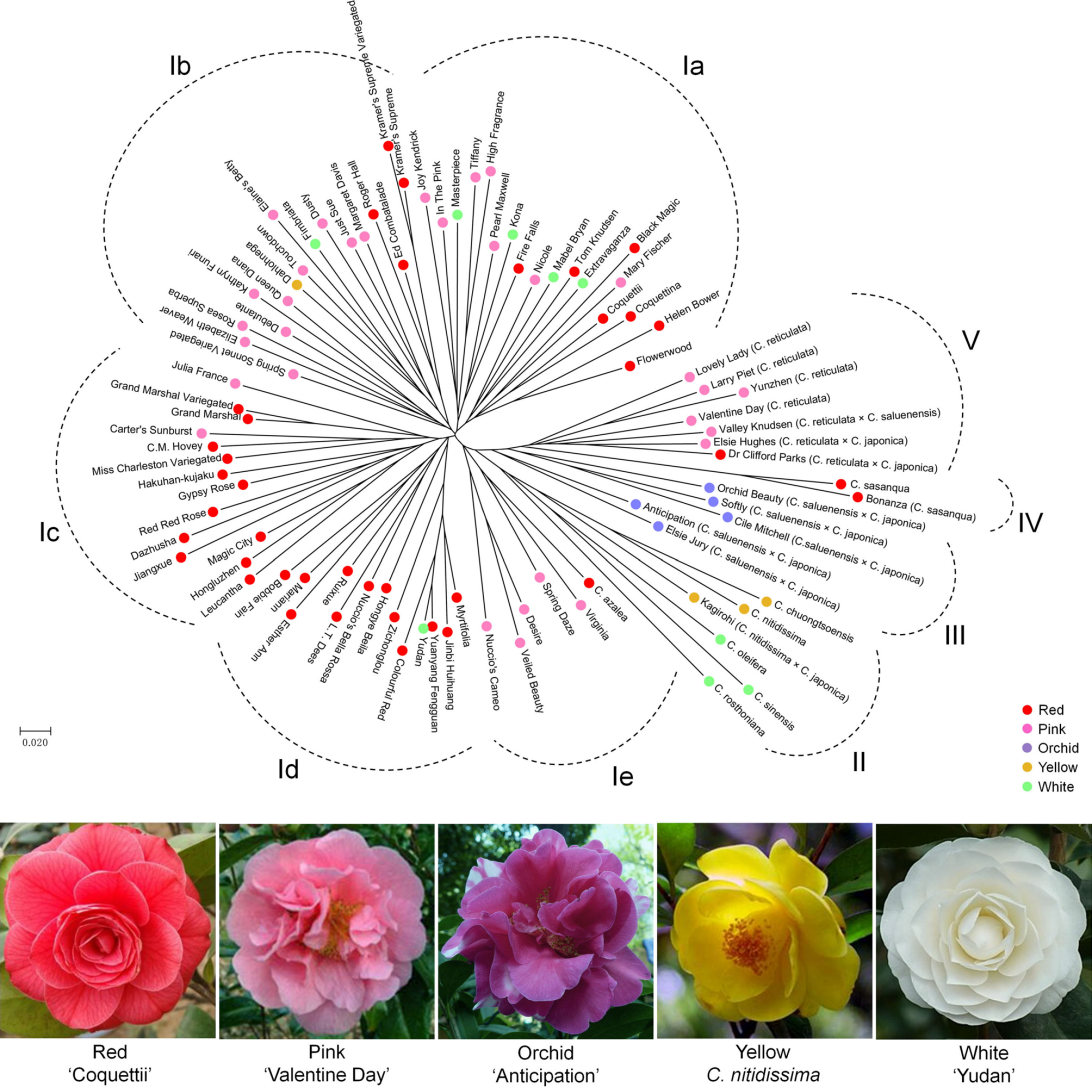
NJ dendrogram showing genetic relationships among the 89 camellia accessions.

It is interesting that when labeling according to flower color, some accessions that were grouped in same cluster or subcluster have similar flower colors. Cluster V (85.7%), subclusters Ib (64.7%) and Ie (83.3%) majority comprised of the accessions with pink flowers. Subclusters Ic (85.7%) and Id (92.3%) mainly contained accessions with red flowers. Cluster II contained accessions with light-colored (yellow or white) flowers. Interestingly, the accessions with orchid flowers were all grouped in cluster III (Fig. 4).

### Principal component analysis (PCA) analysis of natural accessions in the genus *Camellia*

**Figure 5:**
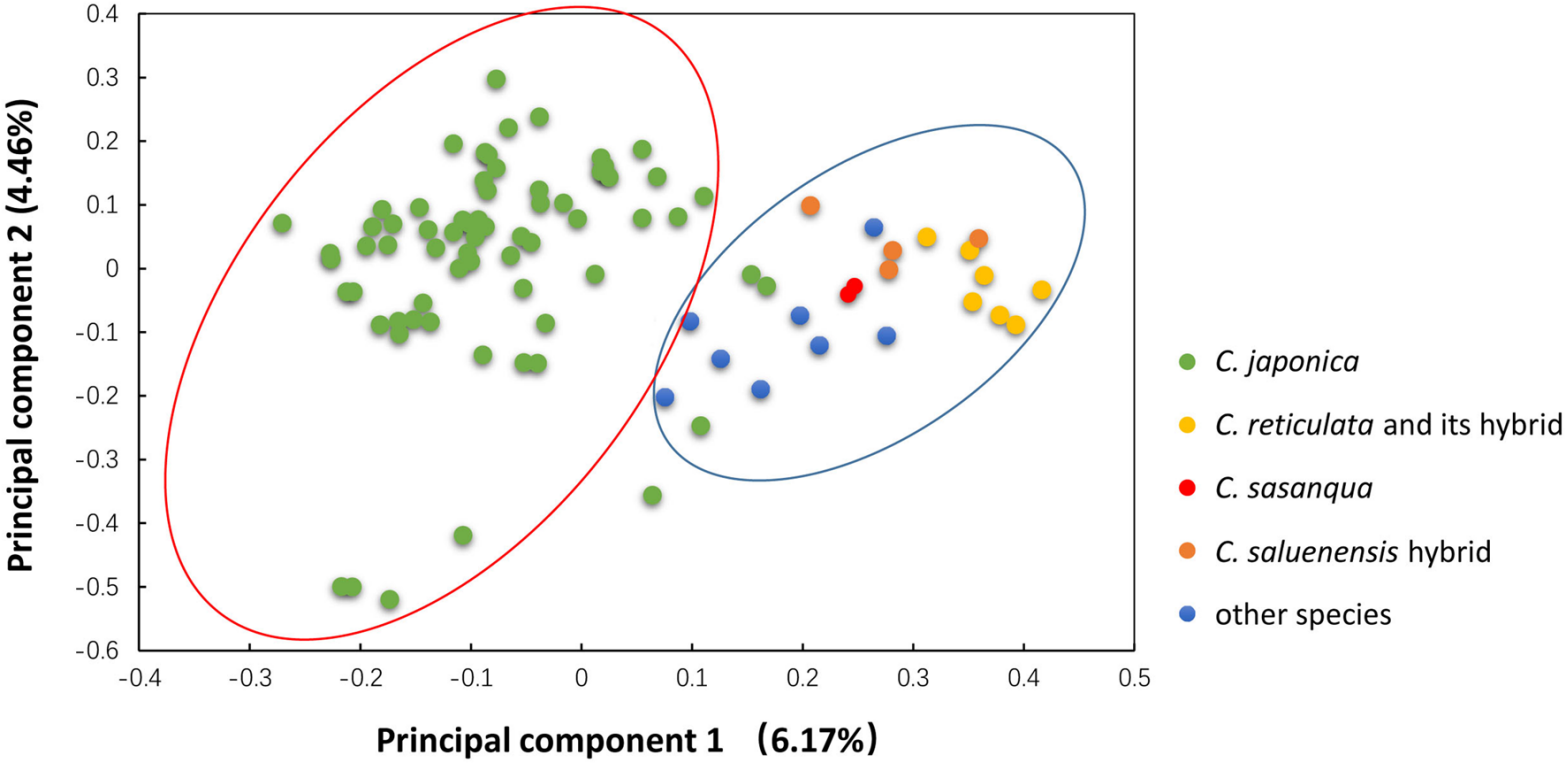
PCA using the first and second eigenvectors identified 89 camellia accessions.

Principal component analysis (PCA) using the first and second eigenvectors identified two major groups (Fig. 5). The eigenvalues of first and second axes were 6.17% and 4.46%, respectively. The accessions from *C. japonica* were noticeably distinct from the accessions that collected from *C. reticulata* and its hybrids, *C. sasanqua*, *C. saluenensis* hybrids, and other species in the genus *Camellia*.

### Genetic structure analyses of selected accessions in the *C. japonica*

**Figure 6:**
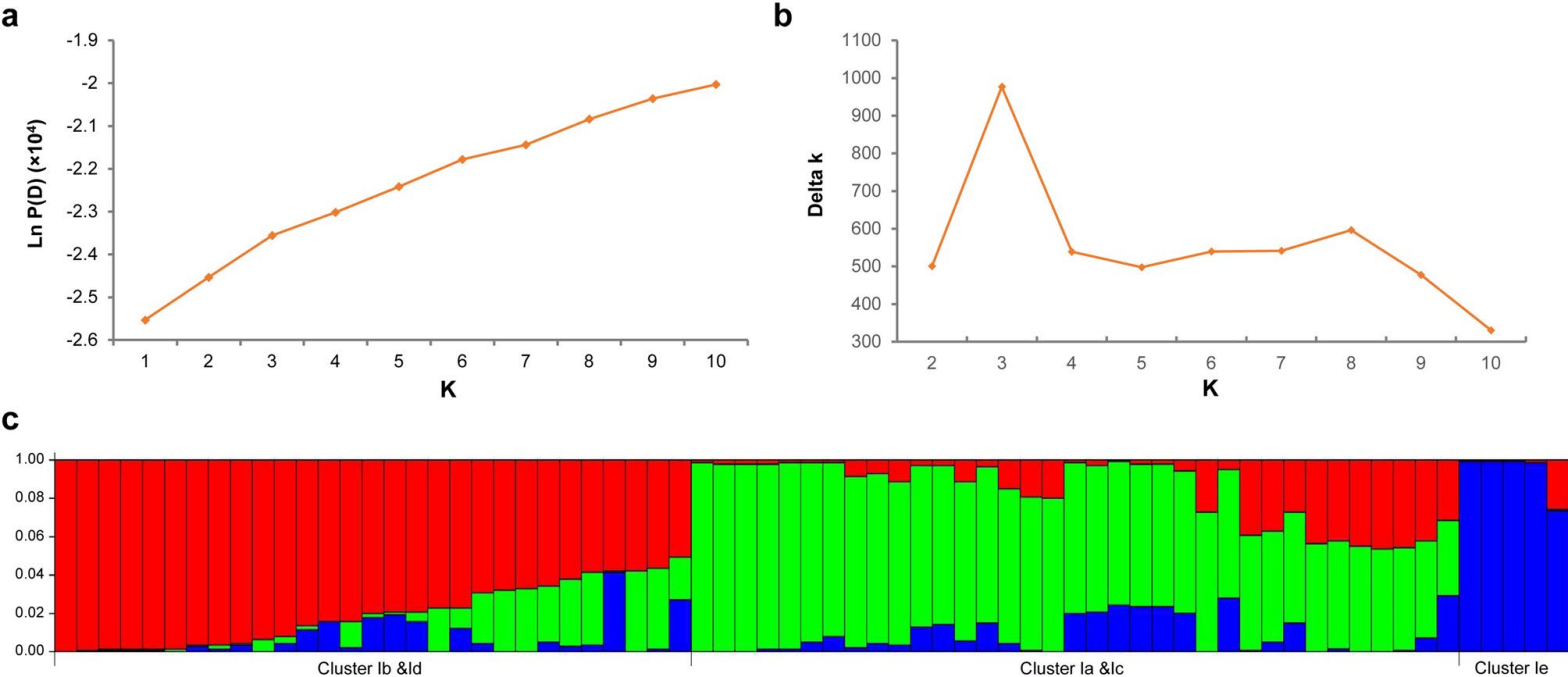
Structure analysis of 69 *C. japonica* accessions. (**a**) Estimated LnP(D) of possible clusters (K) from 1 to 10. (**b**) ΔK based on the rate of change of LnP(D) between successive K. (**c**) Population structure of 69 *C. japonica* accessions. Labels below refer to the cluster number in NJ analysis.

The genetic structure of the 69 accessions from *C.japonica* was analyzed with the Bayesian model-based clustering algorithm implemented in STRUCTURE software. The most likely number of clusters was identified by calculating delta K (ΔK), which is based on the rate of change in the log probability of data between successive K values (K = 1 to K = 10). The peak of the ΔK graph corresponds to the most likely number of populations in the data set. The highest number of ΔK was found at K = 3 (Fig. 6a,b), where all 69 accessions were divided into three main groups: the first group contained cluster Ib and Id that determined in cluster analysis, while cluster Ia and Ic together assigned to group 2, the third group contained cluster Ie that determined in cluster analysis (Fig. 6c). The results from the population structure analysis were generally consistent with the results from the cluster analysis.

## Discussion

SSRs can be divided into genomic SSRs and genic SSRs, and genomic SSRs are usually developed from genomic libraries or random genomic sequences, while genic SSRs are developed from coding regions of the EST or transcriptome sequences^20,24^. Although the discovery of genomic SSR loci using whole genome sequences had been successfully applied in many plant species, such as peanut^29^, pear^30^, sweet potato^31^ and tea plant^13^, genic SSR development based on transcriptome sequences is still fundamental., Genic SSR markers, which are linked to the loci of agronomic phenotypes, are considered more useful for MAS, especially when polymorphic genic SSR markers are identified in breeding lines compared with genomic SSR markers^32^. In previous study, we obtained the high-quality assembly transcriptome sequence of *C. japonica* var. ‘Jiangxue’ based on high-throughput sequencing data^28^, which enabled us to develop SSRs by transcriptome-wide analysis. In this study, 28,854 putative SSRs were identified for approximately 11.74% of the total unigenes. The average frequency of SSRs was 1/4.63 kb, which is similar to those estimated by Ma et al. (1/3.98 kb) and Wu et al. (1/4.99 kb) in tea plants^18,20^ and higher than that estimated by Wen et al. (1/6.5 kb) in oil camellia *C. chekiangoleosa*^24^.

Frequency analysis of motif repeats in *C. japonica* revealed that dinucleotides and trinucleotides were the most abundant types of SSRs and together accounted for 97.59% of all the SSRs identified; tetra-, penta- and hexanucleotides were less abundant and together accounted for 2.35% (Table 1). This tendency of highly abundant di- and trinucleotides was in conformity to reports of several other species in the genus *Camellia*^13,18,20^. Furthermore, the repeat numbers analyze in SSR motifs showed that the distributions of all di-, tri-, tetra-, penta- and hexanucleotides were generally skewed towards fewer repeats (Table 1). Similar tendencies were also found in other species in the genus *Camellia*, such as tea plant^13^ and oil camellia^23^.

Within a certain type of SSR, the frequency of specific repeat motifs may differ. Previous studies on tea plant and other plant species showed that the base composition of SSR motifs is largely biased towards As and Ts^13,33^. Similar results were found in this study: the AG/CT and AT/TA motifs were the most abundant, while the CG/GC motif was very rare among dinucleotide repeats (Supplementary Fig. 1a). Similarly, the motifs AAG/CTT and AAAT/ATTT were the most abundant in tr- and tetranucleotide repeats, respectively, but the percentages of GC-rich repeat motifs were extremely low (Supplementary Fig. 1b,c), indicating that genic SSRs with GC-rich repeats are rare in *C. japonica*. Overall, the abundant genic SSR motifs offer new perspectives for the development of SSR markers in *C. japonica*.

The effectiveness and success of applying SSR markers are considerably dependent on the quality of the markers. In this study, of the 172 newly designed SSR primer pairs, 111 (64.53%) exhibited polymorphism, and the polymorphism ratio was much higher than those obtained in previous studies in the genus *Camellia*^13,18,20,23,34^. The high polymorphism ratio may largely due to the rigorous e-PCR screening that performed in our study. The mean PIC value of the polymorphic markers was 0.59, which was similar to those reported in previous studies on camellia plants^18,23,24^. The high PIC values indicate that these newly developed genic SSR markers are suitable for phylogenetic and genetic diversity analyses and linkage map construction. Moreover, the transferability rates of SSRs in this study were extremely high (from 92.6% to 100%, with an average of 99.38%, Supplementary Table 4), which were higher than those reported in other studies on camellia plants^34,35^. Overall, the high polymorphism ratio and high transferability of the SSR markers developed in this study may be due to the relatively conserved nature of transcriptional sequences. It is interesting that trinucleotide repeats were the most abundant motifs among polymorphic markers (Table 2), although dinucleotide repeats were the most abundant motifs in the *C. japonica* leaf transcriptome (Table 1), implying that the trinucleotide repeat motifs may have a high specificity compared with other repeat motifs in *C. japonica*. Together with the high percentage of trinucleotide repeat motif-containing polymorphic markers among synthesized markers and the high PIC values of the trinucleotide repeat motif-containing polymorphic markers (Table 2), our results in this study indicate that SSR markers developed by trinucleotide repeat motif were the most efficient with high polymorphism and specificity for genetic improvement study in genus *Camellia*.

Highly polymorphic and stable SSR markers are important resources for genetic relationship analysis. Genetic distance measures applied to SSR data can yield useful estimators for phylogenetic relationships among closely related populations, as well as among species, accessions or cultivars^27,30^. In the present study, the NJ dendrogram demonstrated that the accessions from *C. japonica*, *C. reticulata*, *C. sasanqua*, *C. saluenensis* hybrids, and other species were clearly divided into five main clusters, and several accessions known to have similar genetic backgrounds were closely clustered and clearly distinguished with each other in the dendrogram (Fig. 3), indicate the newly developed SSR markers not only can separate the various genus, but also can distinguish the minor/individual variations between accessions. Therefore, the newly developed SSR markers as the core markers had high power for *Camellia* genotyping. Several previous studies showed a significant positive correlation between the genetic distance and geographic distance of ornamental camellia populations^26,27,36^. In the present study, we found that accessions with the similar flower color were grouped together in several clusters/subclusters in the dendrogram (Fig. 4), whereas no regular clustering based on flower type were found in this study (data not shown), implying that flower type may exhibit high probability variations compared with flower color in camellia plants.

The PCA analysis divided all accessions into two main clusters: the first cluster included all *C. japonica* cultivars, while the second cluster contained the accessions that were collected from other species in the genus *Camellia* (Fig. 5). Similar results were obtained in other studies that distinguished *C. japonica* cultivars from other cultivars in the genus *Camellia* using SSR markers^6,26^. A recent study in *C. nitidissima* grouped 96 individuals into 4 subpopulations and found some overlap between subpopulations^27^. In the present study, all the genotypes in *C. japonica* separated and consistently grouped well in the Bayesian model-based genetic structure analysis, although some overlap was also found in subpopulations 1 (clusters Ib and Id) and subpopulation 2 (clusters Ia and Ic) (Fig. 6c). In our study, the selected accessions in *C. japonica* cover almost all flower colors, flower types and breeding approaches (Supplementary Table 5), although the selected accessions in our study do not constitute a classical sexual population, the structure analysis of *C. japonica* may still provide clues for better understanding the origin and evolution of *C. japonica* plants and help us make use of these resources.

## Conclusions

In this study, we developed 28,854 genic SSR markers with a frequency of 4.63 kb from transcriptome sequences of *C. japonica*. A total of 13,323 SSR primers were developed, and among them, 111 were found to be polymorphic in 9 *Camellia* species, which is by far the largest number of SSR markers developed in a single study in *C. japonica* to date. The obtained data also confirmed that the set of 51 SSR markers used in the study revealed a realistic picture of the genetic relationships between 89 camellia accessions. In conclusion, the results of this study will enable further genetic mapping, genetic diversity, and germplasm characterization studies in the genus *Camellia*.

## Methods

### Plant materials and DNA extraction

A total of 89 camellia accessions, planted in the Camellia Germplasm Resource Garden in Wuhan, Forestry and Fruit Tree Research Institute, Wuhan Academy of Agricultural Sciences, were used in the present study. The selected accessions include the original species and cultivars from *C. japonica*, *C. sinensis*, *C. sasanqua*, *C. rosthorniana*, *C. reticulata*, *C. azalea*, *C.nitidissima*, *C. oleifera*, *C. chuongtsoensis*, as well as their interspecific hybrids, which cover the most of the important camellia breeding species in genus Camellia. The selected accessions also cover almost all flower colors, flower types and breeding approaches (Supplementary Table 5). Qingyuan Li is responsible for a formal and more detailed description of each of these accessions. No specific permissions were required for these plant materials, since these studies did not involve endangered or protected species. All experiments including the collection of plant material in this study are in compliance with relevant institutional, national, and international guidelines and legislation.

Genomic DNA was extracted using a Plant Genomic DNA Extraction Kit (TIANGEN, China) following the manufacturer’s instructions. The DNA quality and concentration were determined by electrophoresis in 1% agarose gel and an Agilent 2100 Bioanalyzer (USA). DNA was diluted with sterilized ultrapure water, normalized to 50 ng/μl, and stored at -20 °C until use.

### The identification and annotation of SSR motifs

SSR motifs were identified using MISA^37^. The search parameters were set for the detection of all di-, tri-, tetra-, penta-, and hexanucleotide SSR motifs with a minimum of five repeats. The SSR-containing unigenes were searched using BLASTX against the GO and KEGG^38^ databases (E-value ≤ 1E-5) to retrieve protein functional annotations based on sequence similarity. Plant TFs were predicted using iTAK software^39^.

### Primer design and marker validation

The SSR loci were subjected to primer design using Primer 3 web based software^40^. The parameters were as follows: the length of the primers was in the range of 18–28 bp, with 20 bp as the optimum; the product size range was from 100 to 300 bp; and the melting temperature (Tm) was in the range of 55–65 °C, with 60 °C as the optimum and a maximum Tm difference of 1 °C. All the primer sequences were compared with each other, and primers with more than one copy were deleted to verify that each primer pair amplified only a single SSR. The transcriptome sequences of *C. japonica* were used as templates for SSR markers discovering by using electronic PCR (e-PCR) with the following parameters: 4-bp mismatch, 1-bp gap, and 80–2000 bp product size^41^.

A total of 172 primers were randomly selected from the newly designed primers to first validate PCR and SSR polymorphisms among 10 *C. japonica* accessions (Supplementary Table 2). The primers were synthesized by TSINGKE Biological Technology Co., Ltd. (Beijing, China), and the PCR reagents (buffer, MgCl^2+^, dNTPs and Taq) were purchased from TransGen Biotech Co., Ltd. (Beijing, China). Amplification was programmed as 5 min at 94 °C for initial denaturation; 30 cycles consisting of 30 s at 94 °C for denaturation, 30 s at the annealing temperature (Ta) for annealing, and 45 s at 72 °C for extension; and finally, a 10-min extension step at 72 °C. The PCR products were loaded for electrophoresis in an 8% polyacrylamide gel. Based on the screening results, 51 polymorphic primer pairs were further used to genotype the 89 camellia accessions using the same volume and PCR program.

### Data analysis

Based on the PCR results, a binary matrix was constructed, where the presence of an amplified product was scored as 1 and the absence of the product as 0. Because the templates used in SSR analysis were pooled DNA from 89 plants and more than two alleles per sample were observed, separate bands were treated as individual alleles. The PIC of each locus was calculated using the software PowerMarker version 3.25^42^. The dendrogram was constructed based on Nei’s genetic distances using the NJ method and was viewed by MEGA7^43^.

STRUCTURE software^44^ was used to infer population structure. To identify the number of populations (K) capturing the major structure in the data, a burn-inperiod of 100,000 Markov Chain Monte Carlo (MCMC) iterations was used, with a 100,000run length and an admixture model following Hardy–Weinberg equilibrium. The average ln likelihood value when K changed from 1 to 10 was calculated according to genetic similarity, and each run was replicated five times to ensure consistency of the results.

## Supplementary Information

Supplementary Information 1.

Supplementary Information 2.

## Abbreviations

SSR: Simple sequence repeats
MAS: Marker assisted selection
GO: Gene ontology
KEGG: Kyoto Encyclopedia of Genes and Genomes
TFs: Transcription factors
PIC: Polymorphism information content
NJ: Neighbour-joining
PCA: Principal component analysis
EST: Expressed sequence tag
