# Supplementary material for: Development of genic SSR marker resources from RNA-seq data in Camellia japonica and their application in the genus Camellia
Source: Sci Rep. 2021 May 10;11:9919. doi: 10.1038/s41598-021-89350-w (PMC8110538; doi:10.1038/s41598-021-89350-w)
Supplement: Supplementary file 2 — Supplementary Information 2. [file 41598_2021_89350_MOESM2_ESM.pdf]

Development of genic SSR marker resources from RNA-seq data in Camellia japonica and their application in the genus *Camellia*

Qingyuan Li[1*], Xiaojun Su[3], Huanhuan Ma[3], Kebing Du[2], Min Yang[1,2], Baolin Chen[1], Shao Fu[1], Tianjing Fu[1,2], Conglin Xiang[1,2], Qing Zhao[3], Lin Xu[1*]

[1]Forestry and Fruit Tree Research Institute, Wuhan Academy of Agricultural Sciences, Wuhan 430075, China;

[2]College of Horticulture and Forestry Sciences, Huazhong Agricultural University, Wuhan 430070, China;

[3]College of Plant Science and Technology, Huazhong Agricultural University, Wuhan 430070, China

[*]Corresponding

Lin Xu, xulin@wuhanagri.com and Qingyuan Li, gavin1982@163.com

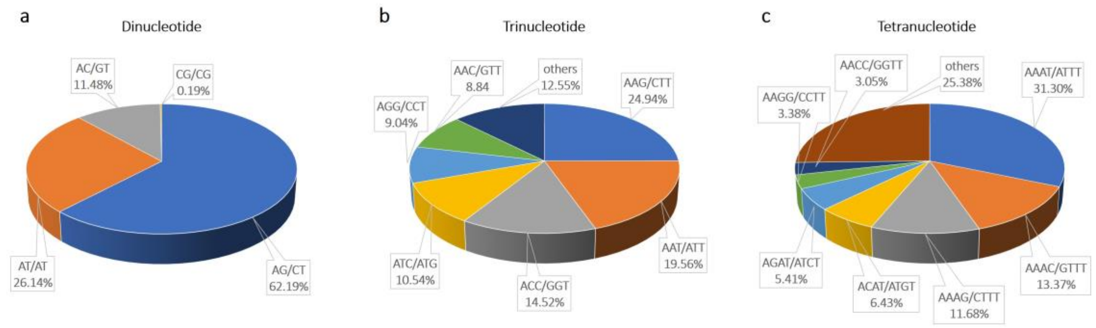

Supplementary Figure 1 Percentages of different SSR motifs among dinucleotide (**a**), trinucleotide (**b**) and tetranucleotide (**c**) repeats in the *C. japonica* transcriptome.
